# Time-series and thematic analyses of clinical utilities and operational issues in early clinical studies of the da Vinci surgical system

**DOI:** 10.1007/s11701-026-03501-7

**Published:** 2026-05-27

**Authors:** Hiroyuki Suzuki, Kaoru Hattori, Kiyotaka Iwasaki

**Affiliations:** 1 Cooperative Major in Advanced Biomedical Sciences, Joint Graduate School of Tokyo Women’s Medical University and Waseda University, Tokyo, 162-8480 Japan; 2 Sony Computer Science Laboratories, Inc, Tokyo Research, Tokyo, 141-0022 Japan; 3 Institute for Medical Regulatory Science, Comprehensive Research Organization, Waseda University, Tokyo, 162-8480 Japan; 4 Waseda Research Institute for Science and Engineering, Waseda University, Tokyo, 162-8480 Japan; 5 Department of Integrative Bioscience and Biomedical Engineering, Graduate School of Advanced Science and Engineering, Waseda University, Tokyo, 162- 8480 Japan; 6 Department of Modern Mechanical Engineering, School of Creative Science and Engineering, Waseda University, Tokyo, 169-8555 Japan

**Keywords:** Robotic surgery, da Vinci, Early clinical adoption, Descriptive evidence synthesis, Temporal evidence accumulation

## Abstract

During early adoption of robotic surgeries, evidence is primarily descriptive, and how such evidence emerges and accumulates over time remains poorly understood. This study introduces a framework for modeling the temporal dynamics of descriptive evidence on device-level utilities and operational issues accumulated during early adoption of the da Vinci system. We employed a three-step approach comprising systematic dataset acquisition, thematic coding of device utilities and operational issues, and quantitative temporal modeling. A PRISMA-guided search of PubMed and Web of Science identified early clinical studies of the da Vinci system from FDA clearance (July 2000) to the first published randomized controlled trial. Two reviewers independently coded descriptive themes, and cumulative occurrence proportions were modeled over time using exponential and logistic regression to characterize the emergence and saturation patterns. Nineteen studies met inclusion criteria, yielding 16 themes (7 utilities, 9 issues) with high inter-rater reliability (agreement rate 93.1%, Cohen’s κ = 0.85). Utilities, particularly instrument dexterity and stereoscopic depth perception, were reported early and reached saturation rapidly, whereas operational issues, including loss of haptic feedback and workflow-related constraints, emerged more gradually and required greater cumulative clinical experience. Time-series modeling demonstrated a clear saturating pattern, with utilities reaching 80% cumulative occurrence at 13.2 months versus 26.0 months for issues. This study presents a framework for modeling how descriptive evidence emerges and matures in early-stage medical device adoption. The observed asymmetry, rapid recognition of utilities versus delayed emergence of operational issues, highlights the importance of structured, continuous synthesis of early clinical evidence.

**Supplementary Information:**

The online version contains supplementary material available at 10.1007/s11701-026-03501-7.

## Introduction

Robotic surgery has transformed minimally invasive surgery since its clinical introduction in the early 2000s. By integrating teleoperated articulated instruments with stereoscopic visualization, robotic systems enable complex maneuvers in anatomically confined spaces that are difficult to perform with conventional laparoscopy [1]. However, ethical constraints and learning-curve effects hinder the feasibility of large randomized controlled trials (RCTs), particularly during early adoption [2]. Thus, early evaluations rely on small case series or observational studies; most evidence is descriptive, limiting early knowledge on effectiveness and safety. While such reports play a critical role in shaping clinical perception and practice, they are rarely examined systematically to understand how evidence emerges and accumulates over time.

The da Vinci^®^ Surgical System (“da Vinci”; Intuitive Surgical, USA) is, as of the mid-2020s, the most widely used surgical robot, and both its clinical utility and operational issues have been reported since early adoption. For example, using stereoscopic endoscopy and robotic instruments, tasks that are complex in conventional laparoscopy—such as vascular anastomosis and organ dissection—have become easier to perform. At the same time, the U.S. Food and Drug Administration (FDA) 2013 MedSun survey report [3] describes operational burdens including complex system setup, staff training requirement, learning-curve effects, and longer operating times; in parallel, high acquisition costs continue to be discussed as a key barrier to global adoption [4]. These issues remain central topics of discussion. Yet the temporal dimension—when these findings first emerged and how they evolved—has not been systematically examined.

Systematic reviews of robotic surgery typically focus on synthesizing quantitative clinical outcomes available at a given time. For example, early systematic reviews of the da Vinci [5, 6] synthesized clinical outcomes—such as operative time, length of stay, complications, and success rates—via meta-analysis. Recent studies show the same pattern: meta-analyses continue to synthesize data on outcomes after prostatectomy in patients with obesity [7], RCT-based cost analyses [8], and learning curves for colectomy [9]. However, these approaches do not capture how descriptive evidence, particularly device-related utilities and workflow-related issues, emerges, recurs, and stabilizes over time in literature. Therefore, the objective of this study is to identify recurring conceptual themes and evaluate whether they reach saturation, consistent with principles of qualitative synthesis.

This study introduces a framework for modeling how descriptive findings on device utilities and operational issues emerge, accumulate, and approach saturation in the published clinical literature. Focusing on the da Vinci system, we combine thematic qualitative analysis with time-series quantitative modeling to analyze reports from FDA clearance of da Vinci Surgical System (July 2000) to the first identified RCT (October 2004). By characterizing the temporal dynamics of theme emergence, this framework is intended to support evidence monitoring during early clinical adoption, inform the prioritization of post-market device improvement, and provide a structured basis for the design of subsequent outcome-based studies, including endpoint selection.

### Methods

**Fig. 1:**
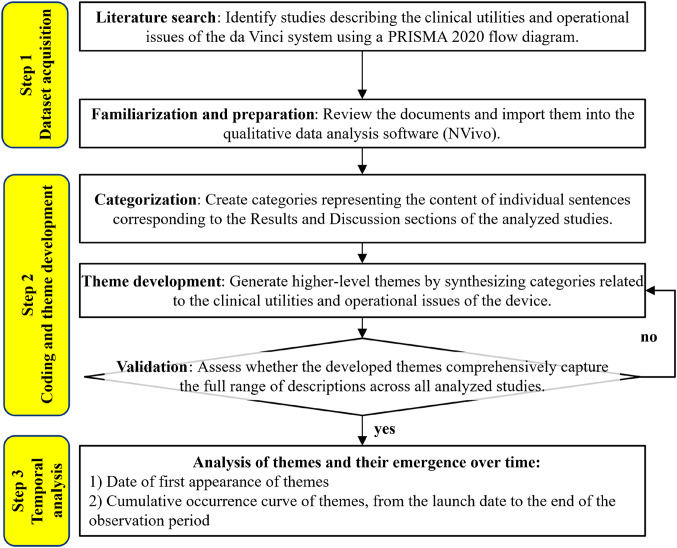
Study workflow. Step 1: Dataset acquisition: Following the PRISMA 2020 flow diagram, we conducted a systematic literature search and screening to assemble the dataset. Step 2: Coding and theme development: We ran thematic analysis on the full text of the Results and Discussion sections and developed themes for utilities and issues. Step 3: Temporal analysis: Using each study’s publication date, we calculated the cumulative occurrence proportion and fit regression models to characterize time trends for the developed themes

This study employed a three-step workflow: (1) systematic dataset acquisition, (2) coding and theme development of clinical utilities and operational issues, and (3) quantitative temporal analysis of theme accumulation (Fig. 1). The study design builds on our previous methodological work [10], which assessed the clinical utilities and operational issues of intraoperative imaging devices using peer-reviewed clinical articles as the analytical dataset. In the present study, this framework was extended by incorporating time-series modeling to quantify how descriptive findings accumulated during the early clinical adoption phase.

#### Step 1. Dataset acquisition

**Fig. 2:**
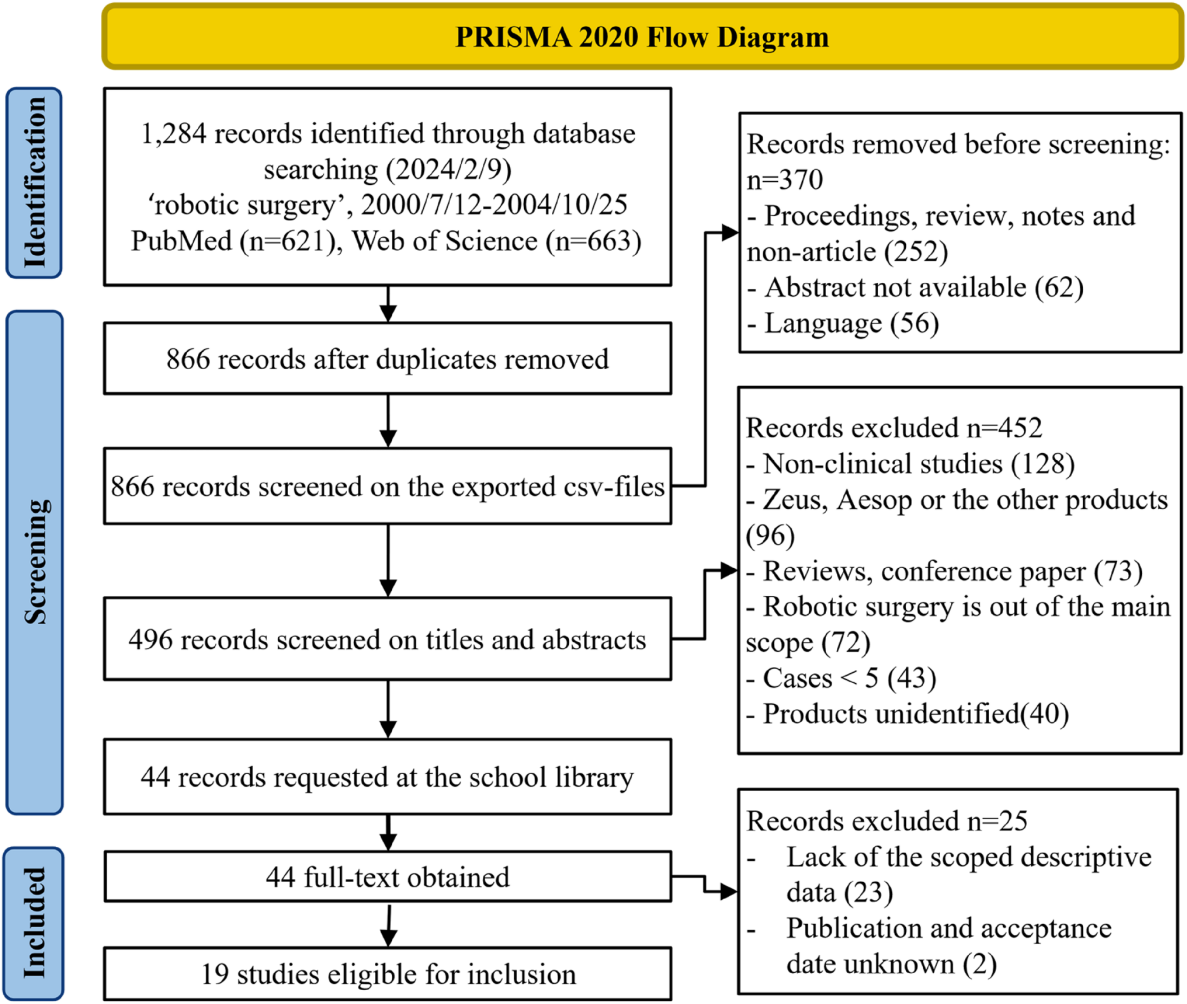
PRISMA 2020 flow diagram. An initial search of PubMed and Web of Science identified 1,284 records reporting early adoption of robotic surgery. After removing duplicates, records were screened against predefined criteria (e.g., language and case count, see Methods), leaving 44 records. Full-text review identified 19 studies within scope

Dataset acquisition followed the PRISMA 2020 Flow Diagram (Fig. 2). Two independent reviewers (H.S., K.H.) conducted the systematic search in PubMed and Web of Science.

The observation window spanned from the day after FDA clearance [11] of the da Vinci system (July 12, 2000) to the day before the publication date of the earliest RCT identified within the prespecified scope (October 25, 2004) [12]. This trial is the earliest da Vinci–based RCT among those included in a systematic review of randomized trials in robot-assisted laparoscopic surgery [13].

To preserve temporal consistency in terminology during the early adoption phase, we used the term “robotic surgery”, which was the predominant descriptor in the literature at that time. Explicit use of da Vinci system was required for inclusion. Records retrieved from both databases were merged in a spreadsheet (Excel, Microsoft, USA) and deduplicated.

Full texts of the retrieved articles were reviewed, and eligibility was determined according to the following criteria: (1) peer-reviewed full-text research articles; (2) English language; (3) surgical robot as the primary focus; (4) human clinical study; (5) sample size ≥ 5; (6) explicit use of the da Vinci system; and (7) inclusion of descriptive statements related to device utilities and operational issues.

Letters, conference proceedings, small case reports (*n* < 5), and review articles were excluded. We excluded small case reports (*n* < 5) to improve the generalizability of the thematic coding of device utilities and operational issues, because very small early reports may be influenced by case selection and learning-curve effects. Because the United States was the first country to approve da Vinci, and to support international accessibility and consistent peer review, we limited the language to English. Articles lacking abstracts or key screening information were also excluded. The independent reviewers resolved disagreements through discussion.

#### Step 2. Coding and theme development

All eligible articles were imported into NVivo (version 14; QSR International, Burlington, MA, USA). One analyst (H.S.) performed sentence-level coding on all sections corresponding to the Results and Discussion. Initial coding was conducted using open, non-hierarchical (free) codes, whereby each sentence was assigned one or more descriptive categories. From these initial categories, those pertaining to device utilities and operational issues in robotic surgery were abstracted into higher-order conceptual themes. Themes were iteratively refined through a generate-review-revise cycle until they consistently spanned the entire dataset, and no further modifications were required. The detailed coding procedures are described in Online Resource 1.

To assess coding reliability, a second analyst (K.H.) independently coded the dataset. A codebook comprising theme names, definitions, and representative examples was first developed by the primary analyst and then used by the second analyst for sentence-level coding in NVivo.

Interpretive consistency between analysts was assessed using the article-level agreement rate as the primary metric. Based on both coding results, we created an Article × Theme presence/absence matrix indicating whether each theme was present in each article. If the number of themes was $\:{N}_{theme}$ and the number of articles was $\:{N}_{article}$; the total number of matrix elements was $\:{N}_{total}\:=\:{N}_{theme}\:\times\:\:{N}_{article}$. Each element was classified as agreement or disagreement between analysts, and the agreement rate was calculated as:

$$\:\text{Agreement rate}=\frac{Agreement}{{Agreement}\;+\;{Disagreement}}$$

A priori threshold of 90% was defined; value below this threshold triggered discussion and revision of theme definitions.

Because agreement rate does not account for chance agreement, Cohen’s κ was also calculated as a chance-corrected index [14]:

$$\:\kappa\:\:=\frac{\left({p}_{o}\:-\:{p}_{e}\right)}{\left(1\:-\:{p}_{e}\right)}$$

where $\:{p}_{o}$ is the observed agreement rate and $\:{p}_{e}$ is the expected agreement by chance.

Agreement rate and κ were calculated using scikit-learn (v1.7.2). κ values were interpreted according to established criteria (< 0.00: Poor agreement, 0.00–0.20: Slight agreement, 0.21–0.40: Fair agreement, 0.41–0.60: Moderate agreement, 0.61–0.80: Substantial agreement, 0.81–1.00: Almost perfect agreement) [14]. If the matrix-level κ was below 0.80, the analysts planned to discuss and revise theme definitions.

#### Step 3. Temporal analysis

To quantify temporal trends in descriptive findings, we derived the cumulative occurrence proportion of each theme identified in Step 2. The analytical procedure is summarized in **Online Resource 2**. For each article, its publication date ($\:Dat{e}_{i}$) was recorded, and the number of newly appearing themes at that $\:Dat{e}_{i}$ ($\:{T}_{new,i}$) was counted. The cumulative number of themes observed up to $\:Dat{e}_{i}$ ($\:{T}_{sum,i}$) was then computed as:

$$\:{\mathrm{T}}_{\mathrm{s}\mathrm{u}\mathrm{m},\mathrm{n}}=\sum_{i=1}^{n}{\mathrm{T}}_{\mathrm{n}\mathrm{e}\mathrm{w},\mathrm{i}}$$

Let $\:{T}_{sum,End}$ denote the cumulative number of themes observed by the publication date of the final article. The cumulative occurrence proportion (%) at each time point was calculated as

$$\begin{aligned}&Cumulative\:occurrence\:proportion\:of\:themes\: (\%)\\&=\frac{{T}_{sum,n}}{{T}_{sum,End}}*100\end{aligned}$$

Within the single article, only the first occurrence of a given theme was counted as an “occurrence.”

We then examined the relationship between cumulative occurrence proportion and two explanatory variables: (1) elapsed time since FDA clearance, and (2) cumulative number of published articles. Regression models were fitted with cumulative occurrence proportion as the outcome and elapsed time or publication count as predictors. Because descriptive findings are expected to accumulate and eventually saturate, we evaluated two candidate models: an exponential model ($\:f\left(t\right)=100(1-{e}^{-\frac{t}{{T}_{c}}}$) and a logistic model ($\:f\:\left(t\right)\:=\:100/(1\:+\:a*{e}^{-bt}))$. Model performance was compared using adjusted R², Akaike Information Criterion (AICc), and root mean square error (RMSE). Regression fitting was performed using SciPy’s curve_fit function (v1.16.2).

Finally, we estimated the elapsed time (or number of publications) required for cumulative occurrence proportions to reach 50%, 80%, and 99.9%, along with corresponding confidence intervals (CIs). Full statistical details and implementation notes are provided in Online Resource 3. The complete analysis code developed for this study is available at the URL provided (see Code availability).

## Results

### Study selection

Figure 2 summarizes the systematic search and screening process in accordance with the PRISMA 2020 Flow Diagram. In February 2024, searches of two databases—PubMed and Web of Science—identified 1,284 records. After deduplication, records were screened by language, abstract availability, and publication type, leaving 496 records. Applying the eligibility criteria (see Methods) yielded 44 records for full-text assessment. Nineteen studies [15–33] met the study scope and were included in the final dataset (Online Resource 4).

### Theme identification and coding reliability

A thematic analysis of the descriptions of the included studies identified 16 distinct themes: 7 utilities (T1-1 ~ 2, T2-1 ~ 2, T3-1 ~ 3) and 9 issues (T4-1 ~ 4, T5-1 ~ 4, T6-1). The full codebook including descriptions, examples, and sources is provided in Online Resource 5.

Two analysts (H.S., K.H.) independently coded theme presence / absence across the $\:{N}_{theme}$ × $\:{N}_{article}$ matrix ((7 + 9)×19 = 304; Online Resource 6 and Online Resource 7). Overall inter-rater consistency was high, with an agreement rate of 93.1% and Cohen’s κ of 0.85 (“Almost perfect”). Both prespecified thresholds were met (agreement rate > 90%, κ > 80%), supporting reliable identification of themes.

### Summary of developed themes

#### T1-1. Dexterity—tasks enabled by instrument dexterity

This theme captured increased maneuverability of robotic instruments in confined operative field and was the most frequently reported utility, appearing in 16 of 19 studies [15, 17–27, 30–33]. In bypass procedures requiring especially fine vascular suturing, such as splenic artery anastomosis and renal artery bypass, several reports noted that suturing and knot tying became easier [15, 17, 24, 33].

#### T1-2. Precision—motion scaling and tremor filtering

Motion scaling and tremor filtering, core functions of teleoperation, were reported in 7 studies [15, 18–20, 22, 32, 33]. These functions were described as facilitating delicate dissection and suturing by suppressing physiological hand tremors.

#### T2-1. Depth perception with stereoscopic vision

Utilities attributable to stereoscopic endoscopy were reported in 12 studies [15, 18, 19, 21, 22, 25–27, 30–33]. Three-dimensional visualization was described as providing depth cues that improved precision in instrument handling [18, 27, 32, 33]. In directional tissue dissection, several reports emphasized that relative three-dimensional spatial relationships among tissues, and between tissues and instruments, were critical information [19, 33].

#### T2-2. Surgeon-controlled viewpoint

A surgeon-controlled viewpoint was reported in 2 studies [21, 32]. Whereas conventional laparoscopy often requires an assistant to anticipate the desired view, robotic systems mount endoscope on a robotic arm and controlled by the primary surgeon, enabling on-demand, stable and precise visualization.

#### T3-1. Improved hand–eye coordination

Improved hand-eye coordination was reported in 5 studies [15, 18, 21, 25, 29]. Several reports described conventional laparoscopic constraints such as mismatches between on-screen motion and instrument motion that impede intuitive positioning, and noted that robotic hand-eye coordination support mitigated these issues.

#### T3-2. Reduced physical strain; neutral posture

Reduced physical load on the surgeon was reported in 5 studies [17, 26, 27, 29, 33]. Unlike conventional procedures that require standing while viewing a distant monitor, robotic surgery allows seated operation at a console with stereoscopic visualization, which was described as reducing fatigue.

#### T3-3. Indexing function

Indexing function, setting the relative position between instrument tip at the patient side and the surgeon’s hand position at the console, was reported in 3 studies [15, 20, 21]. The combination of hand-eye coordination support, an indexing function, and tremor filtering has been described as facilitating tissue manipulation [15].

#### T4-1. Loss of haptic feedback

Loss of haptic feedback was the most frequently reported issues appearing in 16 of 19 studies [15–18, 20–22, 24–27, 29–33]. During suturing and ligation, the absence of force/tension feedback was described as reducing efficiency and requiring training to infer tension visually [15–17, 20, 25, 26, 29, 30]. Five studies additionally noted increased risk of tissue injury attributable to impaired force sensing [18, 25, 27, 32, 33].

#### T4-2. Instrument limitations

Constraints in instrument variety and size were reported in 9 publications [17, 18, 20, 22–25, 27, 30]. Early da Vinci adoption was characterized by a limited instrument lineup; staplers, clip appliers, and suction devices were unavailable, which was considered a drawback compared with conventional laparoscopy, where these tools were already standard [17].

#### T4-3. Device failures

Device malfunctions were reported in 4 studies [18, 25, 26, 33]. Examples included intraoperative detachment of a replaceable hook on an energy device promoting conversion to open surgery [18, 25], or, to conventional laparoscopy [26]. Stereoscopic endoscope failure also led to conversion to conventional laparoscopy [33].

#### T4-4. Control stability

Control-stability limitations inherent to teleoperation were reported in 1 study [15]. Constrained insertion angles were described as causing singularity-related problems; in beating-heart procedures requiring continuous motion, inertia-related effects were reported to induce trajectory overshoot.

#### T5-1. Cumbersome setup pre- and intraoperatively

Setup-related burden associated with a large, multi-arm system was reported in 12 studies [20, 21, 23, 24, 26–33]. Because arm range of motion constrained reachable fields, several reports described the need for case- and procedure-specific adjustments, including arm layout changes, patient repositioning, trocar-site modifications, or additional ports [31, 32].

#### T5-2. Collisions with bulky arms

Collisions between robot arms, or between arms and the patient or other instruments, were reported in 6 studies [15, 18, 22, 24, 29, 33]. Reports described increased likelihood of arm–arm contact in small patients due to short inter-trocar spacing [22], and interference that hindered anastomosis [29].

#### T5-3. Disrupted coordination with assistants

Coordination challenges with assistants were reported in 2 studies [29, 31]. The primary surgeon operates with stereoscopic vision at the console, whereas assistants view a two-dimensional monitor; as a result, assistants may fail to recognize lesions already identified by the surgeon [31]. Large robotic arms were also reported to restrict assistant access to the operating field from both sides of the Table [29].

#### T5-4. Other workflow constraints

Additional workflow limitations and the steep learning curve reported across 6 studies [22, 26, 28, 30, 31, 33]. Limited early experience prompted conversion to conventional laparoscopy in some cases [26]. The size and complexity of the robotic system were also described as making intraoperative troubleshooting more difficult than in conventional procedures [28].

#### T6-1. High costs

High cost relative to conventional approaches were reported in 6 studies [16, 21, 22, 27, 29, 32].

### Theme-source mapping

The presence/absence mapping of all themes across 19 studies is presented in Tables 1 (utilities) and 2 (issues). An asterisk (*) indicates that a study addressed a given theme, and a blank cell indicates absence. Among utilities, the most frequently reported item was improved dexterity (T1-1; 16/19), followed by stereoscopic depth perception (T2-1; 12/19). Among issues, the most frequently reported theme was loss of haptic feedback (T4-1; 16/19), followed by cumbersome setup (T5-1; 12/19 reports).

**Table 1:** Themes-articles mapping (Utilities)

NO.	Study	Publication month	Themes (Utilities)						
			T1-1. Dexterity—tasks enabled by instrument dexterity	T1-2. Precision—motion scaling and tremor filtering	T2-1. Depth perception with stereoscopic vision	T2-2. Surgeon-controlled viewpoint	T3-1. Improved hand–eye coordination	T3-2. Reduced physical strain; neutral posture	T3-3. Indexing function
1	Mohr2001 [15]	2001/05	*	*	*		*		*
2	Rassweiler2001 [16]	2001/07							
3	Pasticier2001 [17]	2001/07	*					*	
4	Ruurda2002 [18]	2002/02	*	*	*		*		
5	Horgan2002 [19]	2002/02	*	*	*				
6	Melfi2002 [20]	2002/05	*	*					*
7	Gutt2002 [21]	2002/07	*		*	*	*		*
8	Gettman2002 [22]	2002/09	*	*	*				
9	Ruurda2003 [23]	2003/02							
10	Giulianotti2003 [24]	2003/07	*						
11	Bentas2003 [25]	2003/08	*		*		*		
12	Talamini2003 [26]	2003/08	*		*			*	
13	Muhlmann2003 [27]	2003/12	*		*			*	
14	Munz2004 [28]	2004/01	*						
15	Desgranges2004 [29]	2004/05					*	*	
16	Bodner2004-1 [30]	2004/05	*		*				
17	Bodner2004-2 [31]	2004/07	*		*				
18	Newlin2004 [32]	2004/07	*	*	*	*			
19	Ayav2004 [33]	2004/09	*	*	*			*	
	Count of *		**16**	**7**	**12**	**2**	**5**	**5**	**3**

An asterisk (“*”) marks studies addressing a given theme; a blank cell indicates the theme is absent

**Table 2:** Themes-articles mapping (Issues)

NO.	Study	Publication month	Themes (Issues)								
			T4-1. Loss of haptic feedback	T4-2. Instrument limitations	T4-3. Device failures	T4-4. Control stability	T5-1. Cumbersome setup pre- and intraoperatively	T5-2. Collisions with bulky arms	T5-3. Disrupted coordination with assistants	T5-4. Other workflow constraints	T6-1. High costs
1	Mohr2001 [15]	2001/05	*			*		*			
2	Rassweiler2001 [16]	2001/07	*								*
3	Pasticier2001 [17]	2001/07	*	*							
4	Ruurda2002 [18]	2002/02	*	*	*			*			
5	Horgan2002 [19]	2002/02									
6	Melfi2002 [20]	2002/05	*	*			*				
7	Gutt2002 [21]	2002/07	*				*				*
8	Gettman2002 [22]	2002/09	*	*				*		*	*
9	Ruurda2003 [23]	2003/02					*				
10	Giulianotti2003 [24]	2003/07	*	*			*	*			
11	Bentas2003 [25]	2003/08	*	*	*						
12	Talamini2003 [26]	2003/08	*		*		*			*	
13	Muhlmann2003 [27]	2003/12	*	*			*				*
14	Munz2004 [28]	2004/01		*			*			*	
15	Desgranges2004 [29]	2004/05	*				*	*	*		*
16	Bodner2004-1 [30]	2004/05	*	*			*			*	
17	Bodner2004-2 [31]	2004/07	*				*		*	*	
18	Newlin2004 [32]	2004/07	*				*				*
19	Ayav2004 [33]	2004/09	*		*		*	*		*	
	Count of *		**16**	**9**	**4**	**1**	**12**	**6**	**2**	**6**	**6**

An asterisk (“*”) marks studies addressing a given theme; a blank cell indicates the theme is absent

### Emergence dynamics of device utilities and operational issues

**Fig. 3:**
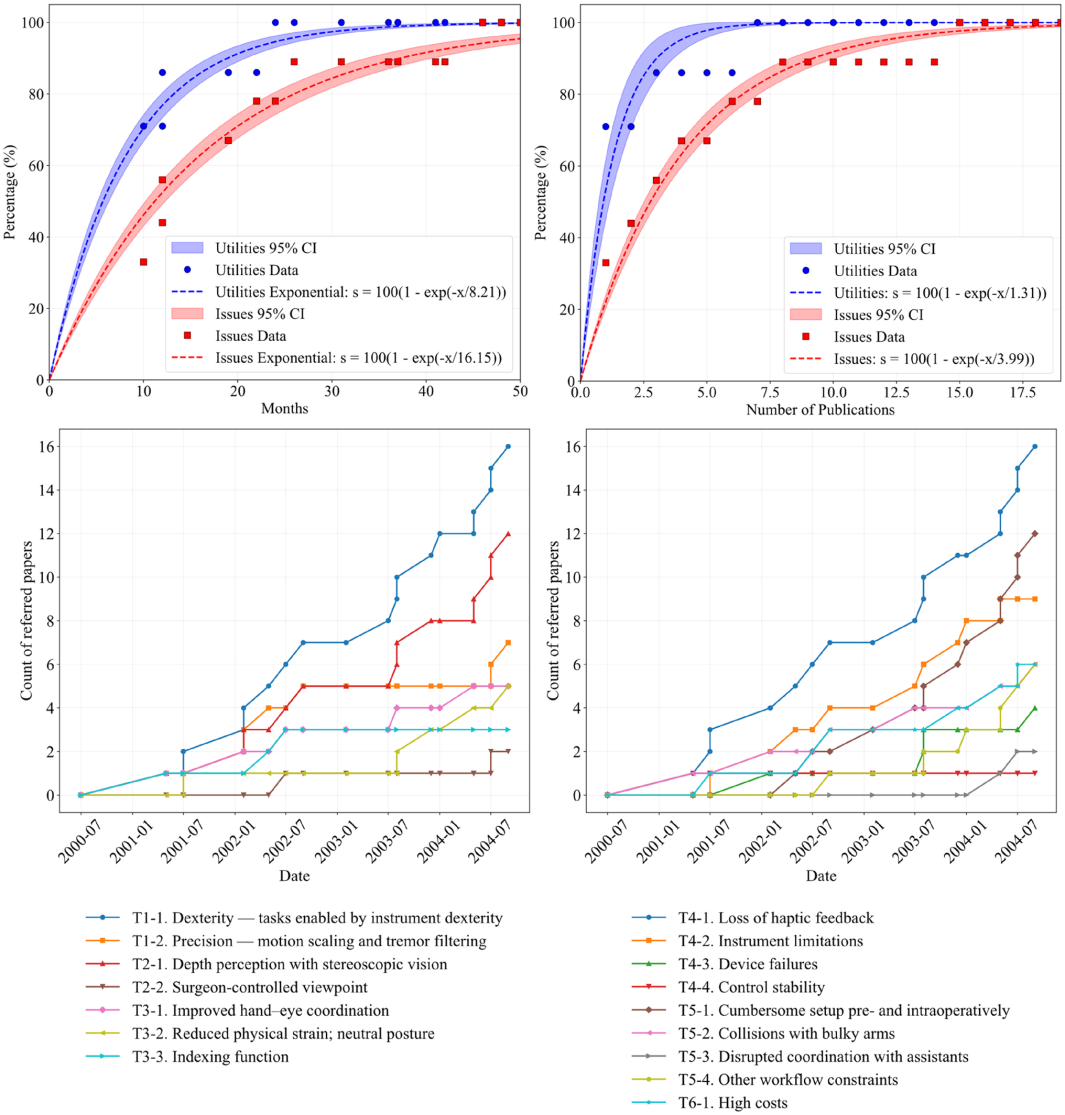
Time-series and publication-count plots for the identified themes of utilities and issues. (Top left) x-axis: months since FDA clearance; y-axis: cumulative occurrence proportion with regression confidence intervals. Circles and squares mark the cumulative occurrence proportion on each publication date. (Top right) x-axis: number of publications; y-axis: cumulative occurrence proportion. (Bottom left) time series of the cumulative count of publications mentioning each utility (T1-1 ~ T3-3). (Bottom right) time series of the cumulative count of publications mentioning each issue (T4-1 ~ T6-1)

Temporal emergence was modeled using cumulative occurrence proportion as the outcome and both months since FDA clearance and publication count as explanatory variables. We compared exponential and logistic regressions; model performance (adjusted R², AICc, and RMSE) in Online Resource 8. Across all analyses, the exponential model consistently provided better fit. For example, for utilities modeled by elapsed months, exponential model outperformed the logistic model (adjusted R², 0.974 vs. 0.938; AICc, 112.8 vs. 132.3; RMSE, 3.7 vs. 5.8). Accordingly, the exponential model was used for subsequent analyses. Under the exponential model, $\:{T}_{c}$ estimates were utilities—8.2 months (95% CI 7.4–9.1), and issues—16.2 months (95% CI 14.6–17.7); when parameterized by publication count, utilities—1.3 publications (95% CI 1.0–1.6) and issues—3.99 publications (95% CI 3.6–4.4). Exponential-model fits are shown in Fig. 3 (top). And logistic-model fits are provided in Online Resource 9.

Figure 3 (bottom) plots time series of the cumulative count of publications that mentions each utilities/issue’s theme. Themes that ultimately appeared in more than half of the included studies—utilities regarding dexterity (T1-1) and depth perception with stereoscopic vision (T2-1) and issues regarding loss of haptic feedback (T4-1) and cumbersome setup pre- and intraoperatively (T5-1)—rose shortly after device introduction. By contrast, surgeon-controlled viewpoint (T2-2) and disrupted coordination with assistants (T5-3) first appeared approximately 2 years and 3.5 years after launch, respectively, indicating delayed recognition.

Table 3 reports the estimated elapsed months and publication counts required to reach cumulative occurrence proportions of 50%, 80%, and 99.9% (with 95% CIs). Utilities reached saturation earlier than issues: 99.9% was reached at 56.7 months for utilities versus 111.5 months for issues. A similar pattern was observed at the 80% threshold (13.2 months for utilities vs. 26.0 months for issues).

**Table 3:** Estimated time and publication count required to reach target proportions

	Target proportion	Period [months, 95% CI]	Number of publications [n, 95% CI]
Utilities	50%	5.7 (5.2 to 6.2)	0.9 (0.7 to 1.1)
	80%	13.2 (11.8 to 14.5)	2.1 (1.6 to 2.6)
	99.9%	56.7 (51.2 to 61.7)	9.0 (7.1 to 11.1)
Issues	50%	11.2 (10.3 to 12.2)	2.8 (2.5 to 3.0)
	80%	26.0 (23.8 to 28.4)	6.4 (5.8 to 7.0)
	99.9%	111.5 (101.7 to 121.9)	27.5 (25.0 to 30.0)

## Discussion

This study introduces a framework for modeling how descriptive evidence on device utilities and operational issues emerges, accumulates, and approaches saturation in the published clinical literature during early clinical adoption. By integrating thematic qualitative analysis with time-series modeling, we demonstrate that device utilities are identified rapidly, whereas operational issues emerge more gradually and require greater cumulative clinical experience. These findings indicate an inherent asymmetry in early evidence formation: favorable, device-centered features tend to be recognized early, while workflow-dependent limitations become apparent only after broader diffusion and increased procedural exposure.

Importantly, this framework is designed to complement, rather than replace, conventional outcome-based evaluation. In early-stage medical device adoption—where randomized evidence is limited or infeasible—descriptive clinical reports represent a primary source of knowledge. By structuring these reports and quantifying their temporal accumulation, the present approach provides a means to support evidence monitoring, identify under-recognized issues, and inform the design of subsequent studies, including endpoint selection and definition of clinically relevant variables.

The observed temporal patterns further support this interpretation. Utilities reached 80% cumulative occurrence within 13.2 months (99.9% at 56.7 months), whereas issues required 26.0 months to reach 80% (99.9% at 111.5 months). Notably, a large proportion of clinically influential observations emerged relatively early, with the time to reach 80% corresponding to approximately one-quarter of the time to reach 99.9%. This suggests that early descriptive evidence, when systematically organized, may provide actionable insights before the availability of high-level evidence.

This framework builds on concepts from both qualitative research and systems analysis. The notion of saturation is informed by cumulative discovery models in fields such as software reliability [34] and by theoretical saturation in qualitative methodologies [35]. The observed saturation patterns for both utilities and issues suggest that temporal analysis of descriptive evidence may serve as a pragmatic indicator of the maturity of the evidence base during early device adoption.

These findings align with contemporary thinking on staged evidence generation. Frameworks such as the IDEAL [36, 37] encourage prospective, structured evaluation from the earliest phases surgical innovation. For example, products launched in the late 2010s to early 2020s—such as Versius^®^ (CMR Surgical) and Hugo^®^ (Medtronic)—have reported evaluations mapped to IDEAL stages [38]. However, designing prospective clinical studies can be challenging for highly innovative technologies—including AI-enabled systems—because novelty of functions and frequent software updates introduce heterogeneity and reduce the utility of “first-in-class” analogs for study design. In such settings, systematically organizing early descriptive findings may help anticipate workflow-related failure modes, prioritize device refinement targets, and guide the transition to outcome-based evaluations.

### Limitations

This study has several limitations. First, the dataset was limited to English-language publications, and the small number of cases in the early adoption phase introduces a risk of geopolitical bias. Second, to preserve temporal consistency in terminology during the early adoption period, the search relied on the then-common term “robotic surgery.” Although broader terminology (e.g., MeSH terms or additional synonyms) may identify additional studies, the major themes were repeatedly observed across independent studies, and the cumulative occurrence curves showed a clear saturating pattern. These findings suggest that the principal conceptual domains were captured, although parameter estimates (e.g., the time constant, Tc) may shift modestly with alternative search strategies. Third, the modeled emergence reflects the timing of publication rather than first clinical awareness. Early reports may preferentially emphasize favorable utilities, introducing potential reporting bias. Therefore, the observed temporal patterns should be interpreted as reflecting the evolution of published evidence rather than the full underlying clinical experience. Forth, although inter-rater agreement and κ indicated robust coding reliability, qualitative analysis retains inherent interpretive components that limit perfect reproducibility. Finally, excluding studies with fewer than five cases may have omitted rare or low-incidence utilities or issues that appear only in early or highly specialized reports. For transparency, we compiled the excluded < 5-case reports and screened them at the title/abstract level; of the 43 excluded reports, 26 were framed as first/initial reports or involved rare/special conditions (see Online Resource 10).

## Conclusions

This study presents a framework for modeling how descriptive evidence on medical device performance emerges, accumulates, and approaches saturation in the published clinical literature during early clinical adoption. By integrating thematic qualitative analysis with quantitative time-series modeling, this study demonstrates that, in the case of the da Vinci Surgical System, device utilities are identified rapidly, whereas operational issues emerge gradually and require greater cumulative clinical experience.

These findings highlight a systematic asymmetry in early evidence formation: favorable, device-centered features tend to be recognized early, while workflow-dependent limitations emerge later. Within a framework that emphasizes temporal consistency and conceptual saturation, structured analysis of early descriptive evidence can provide actionable insights even in the absence of high-level comparative data.

Recognizing this pattern underscores the importance of planned, continuous synthesis of post-market evidence, rather than reliance on early impressions alone. Such an approach may support prioritization of device improvement targets, inform the design of subsequent outcome-based studies, and facilitate safer and more effective integration of novel technologies into clinical practice.

## Electronic Supplementary Material

Below is the link to the electronic supplementary material.

Supplementary Material 1

## Data Availability

No datasets were generated or analysed during the current study.

## Code Availability

The code used to analyze the reliability test results is available at the URL below. https://gitlab.com/hsuzuki_public/articles/surgical_robot_TimeSeries_TA.

## Materials availability

The authors declare that all the data included in this study are available on paper.
