# Supplementary material for: Time-series and thematic analyses of clinical utilities and operational issues in early clinical studies of the da Vinci surgical system
Source: J Robot Surg. 2026 May 27;20(1):538. doi: 10.1007/s11701-026-03501-7 (PMC13216118; doi:10.1007/s11701-026-03501-7)
Supplement: Supplementary file 1 — Supplementary Material 1 [file 11701_2026_3501_MOESM1_ESM.pdf]

Online Resources for: Time-Series and Thematic Analyses of Clinical Utilities and Operational Issues in Early Clinical Studies of the da Vinci Surgical System

Hiroyuki Suzuki, Kaoru Hattori, and Kiyotaka Iwasaki

Overview

The online resources provide supplementary materials supporting the analyses and findings of this study**. Online Resource 1** presents annotated excerpt notes from the included literature, organized by analytic codes, illustrating how categories and themes were developed. **Online Resources 2, 4–7** present tables documenting key calculations and research materials, including cumulative emergence estimates, research lists, the codebook of developed themes, and results from independent coding (utilities and issues). **Online Resource 3** contains additional methodological details, including statistical modeling, parameter estimation, and implementation procedures. **Online Resource 8** additionally reports benchmarking metrics for exponential and logistic model fitting (Adjusted R^2, corrected AIC, and RMSE) in relation to utilities and issues. **Online Resource 9** presents cumulative occurrence plots based on logistic modeling. Finally, **Online Resource 10** shows the study list of excluded reports with fewer than five cases.

Online Resource 1 Notes: Coding procedures

This note organizes excerpts from the literature by codes (analytic categories) and use them to illustrate an example workflow—from category creation to theme development—based on our previous methodological work [1]. The goal is to show how specific device characteristics are discussed in the included clinical studies and how those descriptions inform our themes regarding device-level utilities and operative issues.

For device utilities, we illustrate an example process—from category creation to theme development—using sentences coded under "Dexterity—tasks enabled by instrument dexterity." In Example 1, the text notes that added joints at the instrument tip lets surgeons reproduce traditional open techniques at the console for high-precision tasks, such as vessel dissections. In Example 2, the text describes how wrist articulation helps during procedures involving delicate implants, such as a gastric-band balloon or a pacemaker lead.

Example 1:
"Consequently, surgical precision and accuracy are the real benefits of this technique. At present, the areas of surgery where robotic technique is used to best advantage are those in which the procedures involves a small, deep, fixed operating field and require extreme accuracy (such as vessel dissections). A robotic system with its joint movements allows the surgeon to use his traditional open surgery techniques..." (Melfi et al., 2002, p.867)

Example 2:
"The advantages of a robotic system are related to the articulated instruments that can be moved with seven degrees of freedom. Together with the downscaling of movements, it enables the surgeon to perform more minute and precise actions. Especially for difficult intraoperative settings or for revisional procedures, it may have advantages over conventional laparoscopy. This is of importance in procedures dealing with delicate implants, such as the balloon of the gastric band or the lead of the implantable pacemaker..." (Muhlmann et al., 2003, p.852)

For device issues, we present sentences coded under "Loss of haptic feedback." Example 3 highlights the need to sense gripping force, tension, and instrument position because loss of tactile feedback can damage tissue. Example 4 notes that operating without tactile sensation can pose safety risks during dissection of dense adhesions.

Example 3:
"While our initial experience with the four-arm da Vinci did not involve any perioperative complications such as bleeding or gastric rupture, we did recognize the potential for tissue damage if care is not taken to monitor the position, tension, and grip strength of the instruments". (Newlin et al., 2004, p.123)

Example 4:
"Despite optimal intraabdominal motion control, the lack of tactile sensation may impair the security of meticulous dissection of scar tissue. This led to band removal instead of band exchange, because of suspicion of gastric wall injury that could not be confirmed postoperatively". (Muhlmann et al., 2003, p.853)

Online Resource 2 Table: Calculation of cumulative occurrence proportion

	$Date_{Start}$	$Date_1$	$Date_2$	$Date_3$	…	$Date_i$	…	$Date_{End}$
Time point	Start of observation period	$Article_1$	$Article_2$	$Article_3$	…	$Article_i$	…	End of observation period
No. of newly emerged themes	0	$T_{new,1}$	$T_{new,2}$	$T_{new,3}$	…	$T_{new,i}$	…	$T_{new,End}$
No. of total emerged themes	0	$T_{sum,1}$	$T_{sum,2}$	$T_{sum,3}$	…	$T_{sum,i}$	…	$T_{sum,End}$
Cumulative occurrence proportion	0%	$\frac{T_{sum,1}}{T_{sum,End}}$	$\frac{T_{sum,2}}{T_{sum,End}}$	$\frac{T_{sum,3}}{T_{sum,End}}$	…	$\frac{T_{sum,i}}{T_{sum,End}}$	…	100%

Online Resource 3 Notes: Statistical details and implementation notes

This supplementary note provides an outline of the statistical models, estimation procedures, and implemented source code used in this study. All source code is available at the URL given at the end of this document. Section 1 (S1) describes the calculation of model fit indices based on corrected Akaike Information Criterion (AICc), and S2 describes the calculation based on the adjusted R^2. S3 explains parameter estimation in nonlinear regression models, approximation of the covariance matrix based on the Jacobian, and derivation of confidence intervals (CI) for the parameters. S4 presents the equation-solving procedure for numerically determining thresholds corresponding to achievement rates of 50 %, 80 %, and 99.9 %. It also describes the estimation of confidence intervals for the thresholds that reflect the uncertainty of the estimated parameters. Finally, S5 defines and describes the calculation of the simple agreement rate between raters and Cohen's kappa.

S1. Calculation of AICc

S1-1. Theory

The Akaike information criterion[2] (AIC) is defined in terms of the number of estimated parameters k and the model log-likelihood log(L) as follows:

$$AIC = 2k - 2log(L) \quad (1)$$

In this setting, the number of parameters k used in the AIC includes the error variance σ^2 in addition to the p model parameters (k = p + 1).
The log-likelihood log(L) can be written as follows.

We assume

$$y_i = f(x_i;\theta) + \varepsilon_i,\ \varepsilon_i \sim N(0,\sigma^2) \quad (2)$$

Then, the probability density of each observation is

$$p(y_i|x_i,\theta,\sigma^2) = \frac{1}{\sqrt{2\pi\sigma^2}} \exp\left(-\frac{(y_i - f(x_i;\theta))^2}{2\sigma^2}\right) \quad (3)$$

The joint likelihood represents how likely the observations y_i are when the model prediction $f(x_i;\theta)$ is treated as the mean.

If the ε_i are independent, the likelihood $\mathrm{L}(\theta,\sigma^2)$ is given by the following expression.

$$\mathrm{L}(\theta,\sigma^2)=\prod_{i=1}^{n} p(y_i \mid x_i,\theta,\sigma^2) \tag{4}$$

Thus,

From (3) and (4), defining $e_i(\theta)=y_i-f(x_i;\theta)$, we obtain

$$\log(p(y_i|x_i,\theta,\sigma^2))=-\frac{1}{2}\log(2\pi)-\frac{1}{2}\log(\sigma^2)-\frac{e_i^2}{2\sigma^2} \tag{5}$$

Substituting (5) into (4) gives

$$\log(L(\theta,\sigma^2))=\sum_{i=1}^{n}\log(p(y_i|x_i,\theta,\sigma^2))=\sum_{i=1}^{n}\left[-\frac{1}{2}\log(2\pi)-\frac{1}{2}\log(\sigma^2)-\frac{e_i^2}{2\sigma^2}\right] \tag{6}$$

Collecting terms, the log-likelihood can be written as

$$\log(\mathrm{L})=-\frac{n}{2}\log(2\pi)-\frac{n}{2}\log(\sigma^2)-\frac{1}{2\sigma^2}\sum_{i=1}^{n}e_i^2 \tag{7}$$

Here, evaluating at the maximum likelihood estimates $\theta=\hat{\theta}$, $\sigma^2=\hat{\sigma}^2$, with residuals $e=\hat{e}$, yields

$$\log(\mathrm{L})=-\frac{n}{2}\log(2\pi)-\frac{n}{2}\log(\hat{\sigma}^2)-\frac{1}{2\hat{\sigma}^2}\sum_{i=1}^{n}\hat{e}_i^2 \tag{8}$$

Therefore, from (1), the AIC is

$$\mathrm{AIC}=2k+n\log(2\pi)+n\log(\hat{\sigma}^2)+\frac{1}{\hat{\sigma}^2}\sum_{i=1}^{n}\hat{e}_i^2 \tag{9}$$

where

$$\hat{\sigma}^2=\frac{1}{n}\sum_{i=1}^{n}\hat{e}_i^2 \tag{10}$$

Substituting (10) into (9) gives

$$\mathrm{AIC}=2k+n\log(2\pi)+n\log(\hat{\sigma}^2)+n \tag{11}$$

When n is small relative to k, AIC tends to prefer models with many parameters. To reduce this small-sample bias, we use the corrected Akaike information criterion AICc (corrected Akaike Information Criterion)[2, 3].

$$AICc=AIC+\frac{2k(k+1)}{n-k-1} \tag{12}$$

S1-2. Implementation

Let $\hat{\theta}$ denote the estimated parameter vector, and define the fitted value for the i-th observation as

$$\hat{y}_i=f(x_i;\hat{\theta}) \tag{13}$$

In the code, this prediction is computed as

```
y_pred = model_func(x, *params)
```

Using the sample size n, the number of model parameters p, and the number of parameters k used in the AIC, these quantities are obtained as

```
# Basic statistics
n = len(y)  # Sample size
p = len(params)  # Number of parameters
k = p + 1  # +1 for sigma^2
```

To implement equations (10) and (11), we compute the residuals, the residual sum of squares RSS, and the estimator of σ^2 as

```
residuals = y - y_pred
rss = np.sum(residuals**2)  # Residual sum of squares
 sigma_squared = rss / n
```

Using the simplified form of equation (7), the log-likelihood is evaluated by

```
log_likelihood = -0.5 * n * (np.log(2 * np.pi) + np.log(sigma_squared) + 1)
```

Finally, equations (1) and (12) are coded as

```
aic = 2 * k - 2 * log_likelihood
aicc = aic + (2 * k * (k + 1)) / (n - k - 1)
```

S2. Calculation of adjusted R^2

S2-1. Theory

The coefficient of determination R^2 is given by

$$\mathrm{R}^2 = 1 - \frac{RSS}{SS_{total}} \tag{14}$$

Here, RSS is the residual sum of squares and measures the variation in y not explained by the model. SS_{total} is the total sum of squares and measures the total variation of y around its mean.

$$RSS = \sum_i (y_i - \hat{y}_i)^2 \tag{15}$$

$$SS_{\mathrm{total}} = \sum_i (y_i - \bar{y})^2 \tag{16}$$

Using the sample variance of y, $s_y{}^2$, and the residual variance estimate $\hat{\sigma}^2$, we write

$$s_y{}^2 = \frac{SS_{\mathrm{total}}}{n-1} \tag{17}$$

$$\hat{\sigma}^2 = \frac{RSS}{n-p} \tag{18}$$

Using these relations,

$$R^2_{\mathrm{adj}} = 1 - \frac{\hat{\sigma}^2}{s_y{}^2} = 1 - \frac{RSS}{SS_{total}} \frac{n-1}{n-p} \tag{19}$$

and, substituting (14) into (19),

$$R^2_{\mathrm{adj}} = 1 - (1 - R^2)\frac{n-1}{n-p} \tag{20}$$

which is the usual expression for the R^2_{adj}.

S2-2. Implementation

Equations (14), (16), and (20) are implemented as follows:

```
# Adjusted R² (more appropriate for small samples)
# Calculate R² first for adjusted R² calculation
ss_tot = np.sum((y - np.mean(y))**2)
r2 = 1 - (rss / ss_tot)
adjusted_r2 = 1 - (1 - r2) * (n - 1) / (n - p)
```

S3. Parameter estimation and confidence intervals in the regression model

S3-1. Minimization of RSS and derivation of the covariance

S3-1-1. Formulation

In this study, for the observed data (x_i, y_i) $(i = 1,\ldots,n)$, we assume the nonlinear regression model.

$$y_i = f(x_i;\theta) + \varepsilon_i \tag{21}$$

(1) Nonlinear least squares estimation (RSS minimization)

The residual sum of squares (RSS) is defined as

$$RSS(\theta) = \sum_{i=1}^{n} \left(y_i - f(x_i;\theta)\right)^2 \tag{22}$$

The nonlinear least squares estimator $\hat{\theta}$ is given by

$$\hat{\theta} = \arg\min_{\theta} RSS(\theta) \tag{23}$$

(2) Linearization using the Jacobian

Consider the vector-valued functions

$$f(\theta) = \begin{bmatrix} f(x_1;\theta) \\ \vdots \\ f(x_n;\theta) \end{bmatrix},\ y = \begin{bmatrix} y_1 \\ \vdots \\ y_n \end{bmatrix},\ r(\theta) = y - f(\theta) \tag{24}$$

In a neighborhood of $\hat{\theta}$, we approximate $f(x_i;\theta)$ by its first-order Taylor expansion:

$$f_i(\theta) \approx f_i(\hat{\theta}) + \sum_{j=1}^{p} J_{ij}(\theta_j - \hat{\theta}_j) \tag{25}$$

where

$$J_{ij} = \left.\frac{\partial f_i(\theta)}{\partial \theta_j}\right|_{\theta=\hat{\theta}} \tag{26}$$

Stacking (25) for all $i = 1,\ldots,n$ yields

$$f(\theta) \approx f(\hat{\theta}) + J(\theta - \hat{\theta}) \tag{27}$$

The corresponding residual vector is

$$\begin{aligned} r(\theta) &= y - f(\theta) \\ &\approx y - f(\hat{\theta}) - J(\theta - \hat{\theta}) \end{aligned} \tag{28}$$

Expressing the observations using the true parameter θ_0 and an error vector ε, we write

$$y = f(\theta_0) + \varepsilon \ \Rightarrow\ r(\theta_0) = \varepsilon \tag{29}$$

Substituting $\theta = \theta_0$ into (27) gives

$$f(\theta_0) \approx f(\hat{\theta}) + J(\theta_0 - \hat{\theta}) \ \Rightarrow\ f(\hat{\theta}) \approx f(\theta_0) + J(\hat{\theta} - \theta_0) \tag{30}$$

Thus, from the approximation above we have

$$r(\hat{\theta}) = y - f(\hat{\theta}) \approx (f(\theta_0) + \varepsilon) - (f(\theta_0) + J(\hat{\theta} - \theta_0)) \tag{31}$$

thus,

$$r(\hat{\theta}) \approx \varepsilon - J(\hat{\theta} - \theta_0) \tag{32}$$

In this way, the problem can be written as a least squares problem with the same form as a linear regression model.

(3) Approximation of the covariance of the estimator (pcov)

From the normal equations of linear least squares, setting $\Delta\theta = \hat{\theta} - \theta_0$ gives

$$J^T J \Delta\theta \approx J^T \varepsilon \quad \Rightarrow \quad \Delta\theta \approx (J^T J)^{-1} J^T \varepsilon \tag{33}$$

Here, since $\varepsilon \sim \mathcal{N}(0, \sigma^2 I)$ the covariance matrix of the error vector ε is

$$\mathrm{Cov}(\varepsilon) = \sigma^2 I \tag{34}$$

Therefore,

$$\begin{aligned} \mathrm{Cov}(\Delta\theta) &\approx (J^\mathsf{T} J)^{-1} J^\mathsf{T}\, \mathrm{Cov}(\varepsilon) J (J^\mathsf{T} J)^{-1} \\ &= \sigma^2 (J^\mathsf{T} J)^{-1} \end{aligned} \tag{35}$$

and hence

$$\mathrm{Cov}(\hat{\theta}) \approx \sigma^2 (J^\mathsf{T} J)^{-1} \tag{36}$$

which gives an approximation to the covariance of the nonlinear least squares estimator.

S3-1-2. Implementation

We use the function curve_fit from scipy to estimate the parameters and obtain the covariance matrix. With the default option absolute_sigma=False, the matrix pcov is scaled by the residual variance.

```
# Nonlinear regression
# popt: Optimal parameters like Tc, a, b
# pcov: Covariance of popt
popt, pcov = curve_fit(model_func, x, y, p0=initial_guess)
```

S3-2. Derivation of confidence intervals for parameter estimates

S3-2-1. Theory

For the estimator $\hat{\theta}_j$, we construct a 100($1-\alpha$) [%] confidence interval that is expected to contain the true value $\hat{\theta}_j$:

$$P(\theta_j \in [L_j, U_j]) \approx 1 - \alpha \tag{37}$$

Here, L_j and U_j represent the lower and upper bounds of the interval, respectively. The interval $[L_j, U_j]$ is defined so that, if we repeatedly draw samples from the same population and compute the interval each time, the proportion of intervals that contain θ_j is 1-α.

Using the standard error $SE(\hat{\theta}_j)$, we assume that the standardized statistic $\frac{\hat{\theta}_j - \theta_j}{SE(\hat{\theta}_j)}$, follows a t distribution with ν degrees of freedom. Let $t_{1-\alpha/2,\nu}$ denote the $1-\alpha/2$ quantile of this distribution. Then

$$P\left(-t_{1-\alpha/2,\nu} \le \frac{\hat{\theta}_j - \theta_j}{SE(\hat{\theta}_j)} \le t_{1-\alpha/2,\nu} \right) \approx 1 - \alpha \tag{38}$$

For a 95% confidence interval, we set $\alpha = 0.05$ and obtain

$$P\left(-t_{0.975,\nu} \le \frac{\hat{\theta}_j - \theta_j}{SE(\hat{\theta}_j)} \le t_{0.975,\nu}\right) \approx 0.95 \tag{39}$$

Rearranging both sides gives

$$P\left(\hat{\theta}_j - t_{0.975,\nu} SE(\hat{\theta}_j) \le \theta_j \le \hat{\theta}_j + t_{0.975,\nu} SE(\hat{\theta}_j)\right) \approx 0.95 \tag{40}$$

Defining

$$L_j = \hat{\theta}_j - t_{0.975,\nu} SE(\hat{\theta}_j), \quad U_j = \hat{\theta}_j + t_{0.975,\nu} SE(\hat{\theta}_j) \tag{41}$$

we can rewrite this probability as

$$P\left(\mathrm{L}_j \le \theta_j \le \mathrm{U}_j\right) \approx 0.95 \tag{42}$$

S3-2-2. Implementation

The critical value $t_{\text{crit}} = t_{1-\alpha/2,\nu}$ is obtained from the t distribution as follows:

```
def fit_model_with_confidence(x, y, model_func, initial_guess, confidence_level=0.95):
...
  param_errors = np.sqrt(np.diag(pcov))
...
  # t-value and confidence intervals
  alpha = 1 - confidence_level
  t_crit = stats.t.ppf(1 - alpha/2, stats_dict['dof'])
...
# Parameter confidence intervals
  param_ci_lower = popt - t_crit * param_errors
  param_ci_upper = popt + t_crit * param_errors
```

S4. Estimation of target points corresponding to achievement rates (50%, 80%, 99.9%)

S4-1. Theory

For an achievement rate $p \in \{0.5, 0.8, 0.999\}$ we define the target point x_p as the value that satisfies $f(x_p;\hat{\theta}) = 100p = \text{target}_{\%}$ and estimate x by solving

$$g(x) = f(x;\hat{\theta}) - \text{target}_{\%} = 0 \tag{43}$$

We solve equations of the form

$$f(x;\theta) - \text{target}_{\%} = 0 \tag{44}$$

using the root-finding routines brentq[4] and fsolve[5] from SciPy, and choose between them depending on the properties of the problem. In this study, the model $f(x;\theta)$ is assumed to be monotonically increasing and to have a unique target point within the range of interest. We use the more stable bracketed method brentq as the first option, and fall back to fsolve when no sign change can be found over the search interval. To decide whether brentq can be used, we check that the model behaves monotonically and that the function values at the interval endpoints have opposite signs. When these conditions are satisfied, there is at least one root in the continuous interval.

To obtain confidence intervals for the target points that reflect parameter uncertainty, we proceed as follows.

Step1: Assume that the estimator $\hat{\theta}$ follows a multivariate normal distribution and draw 1000 samples from this distribution.

Step2: For each sampled parameter vector, compute the corresponding perturbed target point x and collect these values.

Step3: From the empirical distribution of these perturbed target points, compute the 95 % confidence interval.

S4-2. Implementation

S4-2-1. Root finding with selective use of brentq and fsolve

For fsolve, we prepared the following initial guesses:

```
initial_guesses=[1, 5, 10, 20, 50]):
```

Each candidate solution obtained by the solvers was checked as follows.

1. Confirm that the solution lies in the allowed range (0 < solution < SOLUTION_MAX_RANGE = 1000)
2. Verify that the residual is sufficiently small (abs(equation(solution)) < SOLUTION_PRECISION = 1e-6)
3. If these checks fail when using fsolve, change the initial guess and rerun the computation.

```
def _solve_equation(equation, initial_guesses=[1, 5, 10, 20, 50]):
    """Helper to solve equation using brentq or fsolve"""
    solutions = []
    for guess in initial_guesses:
        try:
            x_low, x_high = SOLVER_X_LOW, SOLVER_MAX_VALUE
            if equation(x_low) * equation(x_high) < 0:
                solution = brentq(equation, x_low, x_high)
                if 0 < solution < SOLUTION_MAX_RANGE:
                    solutions.append(solution)
            else:
                solution = fsolve(equation, guess)[0]
                if 0 < solution < SOLUTION_MAX_RANGE and abs(equation(solution)) <
SOLUTION_PRECISION:
                    solutions.append(solution)
        except:
            continue
    return solutions
```

S4-2-2. Construction of confidence intervals

Step 1: We assume that the estimator follows a multivariate normal distribution $N(\hat{\theta}, pcov)$ and draw 1000 parameter samples from this distribution:

```
param_samples = np.random.multivariate_normal(params, pcov, CI_SAMPLES)
```

Step 2: For each estimated parameter set and each threshold, we compute the corresponding “perturbed target point.” The variable threshold is given as a proportion between 0 and 1 and is scaled by 100 to match the model output range (0–100):

```
 for threshold in thresholds:
        target_percentage = threshold * 100
        …
        solutions = _solve_equation(equation)
        …
        solution = np.median(solutions)
        …
            for param_sample in param_samples:
                equation_sample = lambda x: float('inf') if x <= 0 else model_func(x, *param_sample) -
target_percentage
        …
                sample_solutions = _solve_equation(equation_sample, [solution])
```

Step3: From the empirical distribution of these perturbed target points, compute the 95 % confidence interval.

```
'ci_lower': np.percentile(threshold_estimates, (alpha/2) * 100),
'ci_upper': np.percentile(threshold_estimates, (1 - alpha/2) * 100)
```

S5. Simple agreement rate and Cohen's kappa

S5.1 Calculation of the simple agreement rate

S5.1.1 Theory

We calculate the simple agreement rate p_o for the classification results provided by raters HS and KH. Because agreement is treated as a binary outcome (True vs. False), p_o is defined as the proportion of agreeing pairs among all valid pairs:

$$p_o = \frac{Agreements\ of\ pairs}{Total\ numbers\ of\ pairs} \tag{45}$$

S5.1.2 Implementation

Equation (45) is implemented as:

```
# Simple agreement rate
agreement_rate = np.mean(hs_valid == kh_valid)
```

Here, hs_valid and kh_valid store the binary ratings (0/1) from raters HS and KH, respectively.

```
def calculate_agreement_metrics(hs_ratings, kh_ratings):
    """Calculate agreement rate and Kappa coefficient"""
    # Exclude missing values
    valid_indices = ~(pd.isna(hs_ratings) | pd.isna(kh_ratings))
    hs_valid = hs_ratings[valid_indices]
    kh_valid = kh_ratings[valid_indices]
```

S5.2 Calculation of Cohen's kappa

S5.2.1 Theory

Cohen's kappa(κ) is defined as the agreement beyond chance, obtained by subtracting the expected agreement by chance[6] from the observed agreement and rescaling by $1-p_e$:

$$\kappa = \frac{p_o - p_e}{1-p_e} \tag{46}$$

where is the simple agreement rate given in (45), and p_e is the agreement expected to occur by chance.

For example, in the binary (0/1) case summarized in the following table, the simple agreement rate p_o is given by

$$p_o = \frac{a+d}{n} \tag{47}$$

For the 2×2 contingency table with cell counts a, b, c and d shown in the following table,

	KH=1	KH=0	Total
HS=1	a	b	a+b
HS=0	c	d	c+d
Total	a+c	b+d	n

- HS assigns category 1: $\frac{a+b}{n}$

- The marginal probability that rater KH assigns category 1: $\frac{a+c}{n}$

- The probability that both raters assign 1 purely by chance: $\left(\frac{a+b}{n}\right)\left(\frac{a+c}{n}\right)$

- The probability that both raters assign 0 by chance: $\left(\frac{c+d}{n}\right)\left(\frac{b+d}{n}\right)$

Therefore, the expected agreement by chance p_e is given by

$$p_e = \left(\frac{a+b}{n}\right)\left(\frac{a+c}{n}\right) + \left(\frac{c+d}{n}\right)\left(\frac{b+d}{n}\right) \tag{48}$$

S5.2.2 Implementation

The kappa coefficient κ is computed using the function cohen_kappa_score from the scikit-learn library:

```
kappa = cohen_kappa_score(hs_valid, kh_valid)
```

References

1. Suzuki H, Tsuboko Y, Tamura M, Masamune K, Iwasaki K. Synthesis of the clinical utilities and issues of intraoperative imaging devices in clinical reports: a systematic review and thematic synthesis. BMC Med Inform Decis Mak. 2025;25:70.
2. Hurvich CM, Tsai C-L. Regression and time series model selection in small samples. Biometrika. 1989;76:297–307.
3. Sugiura N. Further analysis of the data by Akaike's information criterion and the finite corrections: Further analysis of the data by akaike' s. Commun Stat Theory Methods. 1978;7:13–26.
4. Scientific Python Forum. brentq — SciPy v1.16.2 Manual. https://docs.scipy.org/doc/scipy/reference/generated/scipy.optimize.brentq.html. Accessed 7 Dec 2025.
5. Scientific Python Forum. fsolve — SciPy v1.16.2 Manual. https://docs.scipy.org/doc/scipy/reference/generated/scipy.optimize.fsolve.html. Accessed 7 Dec 2025.
6. Landis JR, Koch GG. The measurement of observer agreement for categorical data. Biometrics. 1977;33:159–74.

Source code

https://gitlab.com/hsuzuki_public/articles/surgical_robot_TimeSeries_TA

Online Resource 4 Table: Research lists

No.	Study	Publication date	Design	Samples	Purpose
1	Mohr2001	2001/5	Single-center, case series	148	Summary of the single-institution results with the da Vinci system.
2	Rassweiler2001	2001/7	Single-center, cases	4	The initial experience with tele-surgical laparoscopic radical prostatectomy.
3	Pasticier2001	2001/7	Single-center, case series	5	The early clinical experience with robotically assisted laparoscopic radical prostatectomy.
4	Ruurda2002	2002/2	Single-center, cases multi-surgeon	35	Assessment of the feasibility of robotic surgery for laparoscopic cystectomy
5	Horgan2002	2002/2	Prospective, single-center, case series	10	Summary of the early experiences of the da Vinci system.
6	Melfi2002	2002/5	Single-center, cases, single-surgeon	12	Assessment of the advantages and limitations of robotic thoracoscopic lung surgery.
7	Gutt2002	2002/7	Single-center, cases	11	Feasibility study of robotic surgery for laparoscopic cholecystectomy, fundoplication, and salpingo-oophorectomy in children
8	Gettman2002	2002/9	Single-center, cases	9	Report on a technique and the initial results for Anderson-Hynes pyeloplasty using the da Vinci robotic system.
9	Ruurda2003	2003/2	Single-center, case series, multi-surgeons	10	Evaluation of the procedure time for robotic surgery and identify at what point time loss occurs.
10	Giulianotti2003	2003/7	Single-center, case series, single surgeon	193	A report on the experience with robotic surgery in a large community hospital for general surgery.
11	Bentas2003	2003/8	Single-center, case series, multi-surgeons	40	Report on the initial experience with robot-assisted radical prostatectomy.
12	Talamini2003	2003/8	Prospective, multi-centers, cases, multi-surgeons	201	Prospective evaluation of the safety and usefulness of robotic-assisted surgery.
13	Muhlmann2003	2003/12	Single-center, case series, single surgeon	10	Evaluation of the feasibility of robotic-assisted laparoscopic bariatric surgery and the technical and economic benefits and patient outcomes.
14	Munz2004	2004/1	Cases	6	Report on the surgical technique and preliminary results of the first six cases of robot-assisted sutured rectal resection.

15	Desgranges2004	2004/5	Prospective, single-center, case,	5	Evaluation of the safety and feasibility of robot-assisted aorto-femoral bypass grafting.
16	Bodner2004-1	2004/5	Single center, case series	36	Evaluation of the applicability of robotic surgical robots for common thoracic procedures.
17	Bodner2004-2	2004/7	Single center, case series	14	Report of an institution's experience with a septal mass using the surgical robot.
18	Newlin2004	2004/7	Single center, cases	6	Report on initial experiences with an improved four-arm da Vinci surgical telemanipulation device.
19	Ayav2004	2004/9	Prospective, single center, case series	40	Report on preliminary results of one year of clinical robotic surgery with the da Vinci robotic system.

Online Resource 5 Table: Codebook of the developed themes

Theme	Description	Example sources	Example quotation
**- T1-1. Dexterity — tasks enabled by instrument dexterity**	Wristed articulation at the instrument tip permits fine manipulation in the abdomen, yielding benefits and practice changes compared with conventional surgery.	Bentas2003	*"(2) handling of the laparoscopic tools is greatly facilitated by the degrees of freedom available with the Endowrist instrument technology which enables the surgeon to dissect, suture and tie knots endoscopically as is done in open surgery"*
**- T1-2. Precision — motion scaling and tremor filtering**	Motion scaling and tremor filtering support finer control and steadier execution of demanding tasks.	Horgan2002	*"Additional features facilitating the procedure include a moving camera system, motion scaling, and tremor elimination. These additional features allow the performance of the technically demanding portions of the procedure with extreme precision and accuracy, providing a time-saving advantage."*
**- T2-1. Depth perception with stereoscopic vision**	A stereoscopic endoscope provides magnified 3D vision, helping identify anatomy more safely and accurately.	Horgan2002	*"First, it facilitates the identification and isolation of the ureter very early in the procedure, as a direct result of the magnified three-dimensional view and the articulation of the instrument tips. It also vastly improves the dissection of the renal artery and renal vein, which can be performed with greater safety and accuracy."*

**- T2-2. Surgeon-controlled viewpoint**	Mounted on a robotic arm, the endoscope lets the surgeon control the camera angle directly and rely less on an assistant.	Newlin2004	*"First, it further reduces the surgeon's reliance on assistants during the case. For example, the stereoscopic three-dimensional viewfinder provides a better image that allows the surgeon accurate depth perception. The camera is held steady by the device and controlled tightly by the surgeon rather than by an assistant who must anticipate the surgeon's desired view."*
**- T3-1. Improved hand–eye coordination**	Hand–eye coordination is preserved, which reduces the learning curve and improves performance compared with the fulcrum effect in laparoscopy.	Desgranges2004	*"We have noticed that robotic surgery helped by the da Vinci System does not require a prior training in laparoscopic surgery to obtain the level required for suturing. Unlike standard laparoscopic surgery where the surgeon's hand movements are counterintuitive, the da Vinci System provides natural hand-eye coordination, and as a result, removes a signicant barrier to learning and performing minimal invasive surgery."*
**- T3-2. Reduced physical strain; neutral posture**	Operating from a remote console supports a neutral posture and may lower musculoskeletal injury risk.	Desgranges2004	*"However, the ergonomic environment of the surgeon console should eliminate work-related injuries as it fully supports the body."*
**- T3-3. Indexing function**	Indexing realigns the UI with instrument position, maintaining ergonomic control.	Gutt2002	*"Reindexing is a control feature that allows repositioning of the master arm to an ergonomic position."*
**- T4-1. Loss of haptic feedback**	The loss of tactile feedback can hinder intraoperative judgment and make steps more difficult.	Mohr2001	*"The lack of tactile feedback may lead to impaired decision making in terms of defining the best spot for the anastomosis. If the target vessel is opened in a largely calcified segment, suturing becomes difficult and time consuming."*
**- T4-2. Instrument limitations**	A narrower instrument portfolio or range of motion can limit performance versus conventional tools.	Gettman2002	*"Dissection would be enhanced with the availability of bipolar cautery or the harmonic scalpel."*

**- T4-3. Device failures**	Intraoperative device problems disrupt surgical workflow.	Ruurda2002	*"Mechanical problems occurred in three cases. In these cases the replaceable hook of the electrocautery in strument detached during the procedure. The hook could be removed laparoscopically in two of three cases, but thi s problem resulted in a 4-cm mini laparotomy in one case. This was the single robot-related surgical compli cation."*
**- T4-4. Control stability**	Residual inertia or control instability can degrade precision.	Mohr2001	*"Despite stabilization, there is some residual motion. Due to the mechanical design of the manipulators and end-effectors, residual system inertia may thus become an issue during beating-heart procedures."*
**- T5-1. Cumbersome setup pre- and intraoperatively**	Setup before and during surgery takes time and staff expertise.	Muhlmann2003	*"The set-up of the system is time-consuming. Establishment of the pneumoperitoneum and placement of trocars and robotic arms took a mean of 30 minutes."*
**- T5-2. Collisions with bulky arms**	Bulky arms can collide with the patient or other instruments, complicating the case.	Giulianotti2003	*"Patient positioning is critical because postural changes during different steps of the operation may be required. The robotic cart position and arm settings have to be selected to avoid collisions and to allow assistants to use complementary instruments. The ports must be positioned as in traditional laparoscopy, but usually further apart from each other."*
**- T5-3. Disrupted coordination with assistants**	Arm placement can restrict assistants' access to the field.	Desgranges2004	*"The cumbersome design of the da Vinci does not allow assistants to access both sides of the surgical table."*
**- T5-4. Other workflow constraints**	The system's size and complexity add intraoperative troubleshooting challenges.	Munz2004	*"The second disadvantage is intraoperative trouble shooting. Owing to the huge size of the system any surgical or technical difficulty is more complicated to handle. "*
**- T6-1. High costs**	Costs are substantially higher than for conventional surgery.	Newlin2004	*"The computer-assisted surgical device is not without drawbacks. First, the cost of such a sophisticated piece of technology is beyond the means of most hospitals, surgery centers, or practices."*

Online Resource 6 Table: Independent coding: Utilities

No.	Study	- T1-1. Dexterity — tasks enabled by instrument dexterity		- T1-2. Precision — motion scaling and tremor filtering		- T2-1. Depth perception with stereoscopic vision		- T2-2. Surgeon-controlled viewpoint		- T3-1. Improved hand–eye coordination		- T3-2. Reduced physical strain; neutral posture		- T3-3. Indexing function	
		T1-1_HS	T1-1_KH	T1-2_HS	T1-2_KH	T2-1_HS	T2-1_KH	T2-2_HS	T2-2_KH	T3-1_HS	T3-1KS	T3-2_HS	T3-2_KH	T3-3_HS	T3-3_KH
1	Mohr2001	1	1	1	1	1	1	0	0	1	1	0	0	1	0
2	Rassweiler2001	0	1	0	0	0	1	0	0	0	0	0	0	0	0
3	Pasticier2001	1	1	0	0	0	0	0	1	0	0	1	1	0	0
4	Ruurda2002	1	1	1	1	1	1	0	0	1	1	0	0	0	0
5	Horgan2002	1	1	1	1	1	1	0	1	0	1	0	0	0	0
6	Melfi2002	1	1	1	1	0	0	0	0	0	0	0	0	1	1
7	Gutt2002	1	1	0	0	1	1	1	1	1	1	0	0	1	1
8	Gettman2002	1	1	1	1	1	1	0	0	0	0	0	0	0	0
9	Ruurda2003	0	1	0	0	0	0	0	0	0	0	0	0	0	0
10	Giulianotti2003	1	1	0	0	0	0	0	0	0	0	0	0	0	0
11	Bentas2003	1	1	0	0	1	1	0	0	1	0	0	1	0	0
12	Talamini2003	1	1	0	0	1	1	0	0	0	0	1	1	0	0
13	Muhlmann2003	1	1	0	0	1	1	0	1	0	0	1	1	0	0
14	Munz2004	1	1	0	0	0	0	0	0	0	0	0	0	0	0
15	Desgranges2004	0	0	0	0	0	0	0	0	1	1	1	1	0	0
16	Bodner2004-1	1	1	0	0	1	1	0	0	0	0	0	0	0	0
17	Bodner2004-2	1	1	0	0	1	1	0	0	0	0	0	0	0	0
18	Newlin2004	1	1	1	1	1	1	1	1	0	0	0	0	0	0
19	Ayav2004	1	1	1	1	1	1	0	1	0	0	1	1	0	0

*0 and 1 indicate absence and presence, respectively; the suffixes '_HS' and '_KH' on T1-1~T3-3 mark the initials of the independent analyst.

Online Resource 7 Table: Independent coding: Issues

No.	Study	- T4-1. Loss of haptic feedback		- T4-2. Instrument limitations		- T4-3. Device failures		- T4-4. Control stability		- T5-1. Cumbersome setup pre- and intraoperatively		- T5-2. Collisions with bulky arms		- T5-3. Disrupted coordination with assistants		- T5-4. Other workflow constraints		- T6-1. High costs	
		T4-1_HS	T4-1_KH	T4-2_HS	T4-2_KH	T4-3_HS	T4-3_KH	T4-4_HS	T4-4_KH	T5-1_HS	T5-1_KH	T5-2_HS	T5-2_KH	T5-3_HS	T5-3_KH	T5-4_HS	T5-4_KH	T6-1_HS	T6-1_KH
1	Mohr2001	1	1	0	0	0	0	1	1	0	0	1	1	0	0	0	0	0	0
2	Rassweiler2001	1	1	0	1	0	0	0	0	0	0	0	0	0	1	0	0	1	1
3	Pasticier2001	1	1	1	1	0	0	0	0	0	0	0	0	0	0	0	0	0	0
4	Ruurda2002	1	1	1	1	1	0	0	0	0	0	1	1	0	0	0	0	0	0
5	Horgan2002	0	0	0	0	0	0	0	0	0	0	0	0	0	0	0	0	0	0
6	Melfi2002	1	1	1	1	0	0	0	0	1	1	0	0	0	0	0	0	0	0
7	Gutt2002	1	1	0	1	0	0	0	0	1	1	0	0	0	0	0	0	1	1
8	Gettman2002	1	1	1	1	0	0	0	0	0	0	1	1	0	0	1	1	1	1
9	Ruurda2003	0	0	0	0	0	0	0	0	1	1	0	0	0	0	0	0	0	0
10	Giulianotti2003	1	1	1	1	0	0	0	0	1	1	1	1	0	0	0	1	0	0
11	Bentas2003	1	1	1	1	1	0	0	0	0	0	0	0	0	0	0	0	0	0
12	Talamini2003	1	1	0	0	1	1	0	0	1	1	0	0	0	0	1	1	0	0
13	Muhlmann2003	1	1	1	1	0	0	0	0	1	1	0	0	0	0	0	0	1	1
14	Munz2004	0	0	1	1	0	0	0	0	1	1	0	0	0	0	1	1	0	0
15	Desgranges2004	1	1	0	0	0	0	0	0	1	1	1	1	1	1	0	0	1	1
16	Bodner2004-1	1	1	1	1	0	0	0	0	1	1	0	0	0	1	1	1	0	0
17	Bodner2004-2	1	1	0	0	0	0	0	0	1	1	0	1	1	1	1	1	0	0
18	Newlin2004	1	1	0	0	0	0	0	0	1	1	0	0	0	0	0	0	1	1
19	Ayav2004	1	1	0	0	1	0	0	0	1	1	1	0	0	0	1	1	0	0

*0 and 1 indicate absence and presence, respectively; the suffixes '_HS' and '_KH' on T4-1~T6-1 mark the initials of the independent analyst.

Online Resource 8 Table: Benchmark of model fitting (Adjusted R^2, corrected AIC and RMSE) for exponential and logistic models in relation to utilities and issues.

Indicators	Model	Period (Utilities)	Period (Issues)	Publications (Utilities)	Publications (Issues)
Adjusted R^2	Exponential	0.974	0.959	0.924	0.969
	Logistic	0.938	0.946	0.842	0.920
AICc	Exponential	112.8	127.3	134.5	121.6
	Logistic	132.3	134.7	150.9	142.6
RMSE	Exponential	3.7	5.3	6.4	4.6
	Logistic	5.8	6.1	9.2	7.5

Online Resource 9 Fig: Cumulative occurrence plots (Logistic model based)

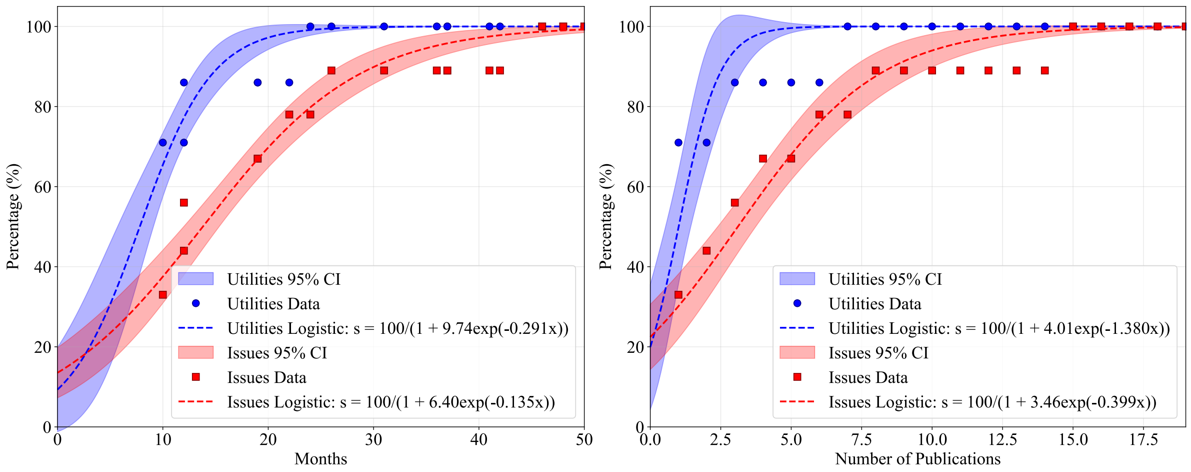

Online Resource 10. Study list of excluded reports with fewer than five cases (N<5) screened at the abstract level.

No.	DB	PMID	Year	Title	Authors	N	Cases	Categories Pilot/First-in-human report/Rare cases	Excerpt
1	PubMed	11061367	2000	Totally endoscopic atrial septal defect closure using robotic techniques: report of two cases	L Torracca et al.	2	Totally endoscopic atrial septal defect (ASD) closure	First-in-human / First report	"Open-heart closure of an ASD without opening the chest has never been previously reported".
2	PubMed	11444320	2001	Robotic Nissen fundoplication: alternative surgical technique for the treatment of gastroesophageal reflux disease	W H Chapman et al.	1	Nissen fundoplication	First-zin-human / First report	"The first robotic Nissen fundoplication using the da Vinci robotic surgical system …"
3	PubMed	11569517	2001	Computer-assisted robotic heller myotomy: initial case report	W S Melvin et al.	1	Heller myotomy	Not applicable	-
4	PubMed	11727154	2001	Totally endoscopic Nissen fundoplication with a robotic system in a child	D D Meininger et al.	1	Nissen fundoplication	First-in-human / First report Special condition	"To our knowledge, this is the first such case to be reported. In addition to standard monitoring, we used invasive blood pressure monitoring during the 282-min period of general anesthesia".
5	WoS	11435858	2001	Robot assisted laparoscopic nephrectomy	B Guillonneau et al.	1	Nephrectomy	First-in-human / First report	"To our knowledge we report the first robot assisted, laparoscopic nephrectomy in a human".
6	WoS	11515911	2001	Computer-enhanced totally endoscopic sequential arterial coronary artery bypass	S Dogan et al.	2	Coronary artery bypass	Not applicable	-
7	PubMed	11854750	2002	A surgeon's obligations when performing new procedures	James W Jones et al.	2	Abdominal aneurysmectomies	Ethics / Commentary	"Where might we most expect to see an ethical problem in the

									surgeon's management of the new case?"
8	PubMed	11912372	2002	Robotic assisted kidney transplantation: an initial experience	Andrá Hoznek et al.	1	Kidney transplantation	First-in-human / First report	"We investigated the feasibility of robotic assisted kidney transplantation".
9	PubMed	11948302	2002	Robotic-assisted adrenalectomy for adrenal incidentaloma: case and review of the technique	James A Young et al.	1	Resection of a left adrenal mass	Rare disease / Rare pathology	"Pathology demonstrated a rare adrenal oncocytoma".
10	PubMed	12031759	2002	Anesthetic implications of the addition of an operative robot for endoscopic surgery: a case report	K Gage Parr et al.	1	Nissen fundoplication	Not applicable	-
11	PubMed	12227912	2002	Laparoscopic transperitoneal adrenalectomy using a remote-controlled robotic surgical system	Wassilios Bentas et al.	4	Adrenalectomy	First-in-human / First report	"We demonstrate the feasibility of performing laparoscopic adrenalectomy exclusively by using robotic telepresent technology from a remote workstation".
12	PubMed	12422124	2002	Robot-assisted laparoscopic aortobifemoral bypass for aortoiliac occlusive disease: a report of two cases	Willem Wisselink et al.	2	Aorto-bifemoral bypass	First-in-human / First report	"To our knowledge, this is the first report on robot-assisted laparoscopic aortobifemoral bypass in the world literature".
13	PubMed	12475680	2002	Robotic-assisted laparoscopic adrenalectomy	Mihir M Desai et al.	2	Laparoscopic adrenalectomy	First-in-human / First report	"We present, to our knowledge, the initial 2 cases of robotic-assisted laparoscopic adrenalectomy in humans".
14	PubMed	12538146	2002	Totally endoscopic atrial septal defect repair with robotic assistance	Michael Argenziano et al.	1	Atrial septal defect (ASD) repair	First-in-human / First report	"This represents the first U.S. application of robotic technology

									for totally endoscopic open-heart surgery".
15	PubMed	17669972	2002	Total robotic-enhanced pericardiectomy for effusive pericarditis	O Reuthebuch et al.	1	Endoscopic pericardiectomy	Not applicable	-
16	WoS	12473897	2002	Telerobotic-assisted laparoscopic right and sigmoid colectomies for benign disease	Philip A Weber et al.	2	Colectomies	First-in-human / First report	"In this article, we present the first two reported cases of telerobotic-assisted laparoscopic colectomies performed on March 6 and 8, 2001".
17	PubMed	12505914	2003	Anesthesia for robotic repair of the mitral valve: a report of two cases	Nutan Mehta et al.	2	Mitral valve repair	Not applicable	-
18	PubMed	12535381	2003	Robot-assisted thoracoscopic resection of a benign mediastinal neurogenic tumor: technical note	Jelle P Ruurda et al.	1	Thoracoscopic removal	First-in-human / First report	"The first case report of robot-assisted thoracoscopic removal of a benign neurogenic tumor in the thorax is presented".
19	PubMed	12676019	2003	Robotic resection of pancreatic neuroendocrine tumor	W S Melvin et al.	1	Pancreatic resection	First-in-human / First report	"Here we report the first known case of pancreatic resection with use of a computer-assisted, or robotic, surgical device".
20	PubMed	12822644	2003	Resection of a symptomatic pericardial cyst using the computer-enhanced da Vinci Surgical System	Matthew D Bacchetta et al.	1	Symptomatic pericardial cyst	Not applicable	-
21	PubMed	12853789	2003	Laparoscopic radical prostatectomy: description of the extraperitoneal approach using the da Vinci robotic system	Matthew T Gettman et al.	4	Radical prostatectomy	First-in-human / First report	"We developed and assessed the feasibility of extraperitoneal laparoscopic radical prostatectomy performed using the da Vinci (Intuitive Surgical,

									Mountain View, California) robotic system".
22	PubMed	12913728	2003	Robotic assisted laparoscopic sural nerve grafting during radical prostatectomy: initial experience	Jihad H Kaouk et al.	3	Sural nerve grafting after radical prostatectomy	First-in-human / First report	"We describe a novel technique of laparoscopic sural nerve grafting after radical prostatectomy using the da Vinci (Intuitive Surgical, Mountain View, California) robot".
23	PubMed	12932932	2003	Robotic-assisted laparoscopic radical cystectomy and intra-abdominal formation of an orthotopic ileal neobladder	W-D Beecken et al.	1	Radical cystectomy and intra-abdominal formation	First-in-human / First report	"We describe our surgical technique in the worldwide first attempt to perform a robotic-assisted laparoscopic radical cystectomy and completely intra-abdominal formation of an orthotopic neobladder".
24	PubMed	14642031	2003	Laparoscopy-assisted robotic radical cystoprostatectomy with ileal conduit urinary diversion for muscle-invasive bladder cancer: initial two cases	Paulos Yohannes et al.	2	Radical cystoprostatecto my	Not applicable	-
25	PubMed	14714773	2003	Pericardial patch closure of an atrial septal defect using endoscopic robotic technology	Justin Resley et al.	1	Full cardiopulmonary bypass (CPB)	Not applicable	-
26	PubMed	14722003	2003	Endoscopic computer-enhanced mediastinal mass resection using robotic technology	Jeffrey A Morgan et al.	2	Resection of mediastinal masses	Not applicable	-
27	PubMed	14722004	2003	Thoracoscopic lobectomy using robotic technology	Jeffrey A Morgan et al.	1	Thoracoscopic lobectomy	Not applicable	-

28	PubMed	17669992	2003	Thoracoscopic thymectomy with the 'da Vinci' surgical system in patient with myasthenia gravis	Federico Rea et al.	1	Myasthenia gravis	Not applicable	-
29	WoS	NONE	2003	Splenic artery aneurysm - Robotic resection and vascular reconstruction	Andolfi, E; Savarese, L; Gentile, E; Coratti, A; Giulianotti, PC	1	Splenic artery aneurysms	Rare disease / Rare pathology	"Splenic artery aneurysms are rare…"
30	PubMed	14625762	2004	Robot-assisted choledochotomy: feasibility	G Roeyen et al.	1	Clearing stones from the common bile duct	First-in-human / First report	"As far as we could ascertain, we report the first case of a laparoscopic choledochotomy with the assistance of this robotic system".
31	PubMed	14751347	2004	Feasibility of robot-assisted totally intracorporeal laparoscopic ileal conduit urinary diversion: initial results of a single institutional pilot study	K C Balaji et al.	3	Totally intracorporeal laparoscopic ileal conduit urinary diversion (TLIC)	Not applicable	-
32	PubMed	14760482	2004	Robot-assisted laparoscopic aortic reconstruction for occlusive disease-a case report	Lois A Killewich et al.	1	Aortic reconstruction for occlusive disease	First-in-human / First report	"The authors report the first case of an aortic reconstruction for occlusive disease performed using the da Vinci system".
33	PubMed	14973683	2004	Robot-assisted mediastinal parathyroidectomy	C Profanter et al.	1	Resection of a mediastinal parathyroid adenoma in the	Not applicable	-

							aorto-pulmonary window		
34	PubMed	14992741	2004	Robot-assisted radical cystectomy and urinary diversion in female patients: technique with preservation of the uterus and vagina	Mani Menon et al.	3	Radical cystectomy and urinary diversion in women	First-in-human / First report	"To the best of our knowledge, this is the first case series of robot-assisted radical cystectomy and urinary diversion in women".
35	PubMed	15026895	2004	Robotic-assisted thoracoscopic resection of esophageal leiomyoma	E Elli et al.	2	Resection of esophageal tumors	First-in-human / First report<br>Rare disease / Rare pathology	"We present the first reported robotic-assisted thoracoscopic resection of two patients…"<br><br>"Esophageal leiomyoma represents 70-80% of all benign esophageal tumors but only approximately 0.5% of all esophageal neoplasms…".
36	PubMed	15138099	2004	Totally endoscopic coronary artery bypass on the beating heart in Jehovah's Witness and HIV patients: case report	Roberto Casula et al.	2	Direct coronary artery bypass	Special condition	"In the first case, a Jehovah's Witness patient, blood transfusion was not an option; in the second case, a human immunodeficiency virus (HIV)-positive patient …"
37	PubMed	15259586	2004	The da Vinci robot in right adrenalectomy: considerations on technique	Annibale D'Annibale et al.	1	Right adrenalectomy for a right adrenal mass	Not applicable	-
38	PubMed	15296917	2004	Totally endoscopic robotic-guided pulmonary veins ablation: an alternative method for the treatment of atrial fibrillation	Gino Gerosa et al.	1	Epicardial pulmonary veins ablation	Not applicable	-

39	PubMed	15320575	2004	First Italian robot-enhanced coronary bypass	Gino Gerosa et al.	1	Totally endoscopic coronary artery bypass	First-in-human / First report	"This case report presents the first totally endoscopic coronary artery bypass performed ..."
40	PubMed	15450591	2004	Totally endoscopic off-pump bilateral internal thoracic artery bypass grafting	Fadi Farhat et al.	1	Totally endoscopic off-pump bilateral ITA grafting	First-in-human / First report	"We report the first case of a totally endoscopic off-pump bilateral ITA grafting in a 58-year-old patient".
41	PubMed	15464454	2004	Robotically-assisted left atrial fibrillation ablation and mitral valve repair through a right mini-thoracotomy	Gil Bolotin et al.	1	Left atrial ablation and mitral valve repair	Not applicable	-
42	PubMed	15554274	2004	Making the transition from standard gynecologic laparoscopy to robotic laparoscopy	Jennifer L Ferguson et al.	4	Bilateral tubal ligation	First-in-human / First report	"To obtain robotic credentialing and gain experience with the robotic system, the surgeons first went through robotic training, then 4 women desiring permanent sterilization had robotically assisted laparoscopic bilateral tubal ligations performed, using the Parkland method".
43	PubMed	15769680	2004	Robot-assisted off-pump minimally invasive reoperative coronary artery bypass grafting: case report	Timothy P Martens et al.	1	Reoperative coronary artery bypass grafting (CABG)	Not applicable	-
